# Petroleum-derived naphthenic acids disrupt hormone-dependent sexual behaviours in male Western clawed frogs

**DOI:** 10.1093/conphys/coac030

**Published:** 2022-05-17

**Authors:** Wo Su Zhang, Elizabeth J Farmer, Daniella Muhanzi, Vance L Trudeau

**Affiliations:** Department of Biology, University of Ottawa, Ottawa, ON K1N 6N5, Canada; Department of Biology, University of Ottawa, Ottawa, ON K1N 6N5, Canada; Department of Biology, University of Ottawa, Ottawa, ON K1N 6N5, Canada; Department of Biology, University of Ottawa, Ottawa, ON K1N 6N5, Canada

**Keywords:** endocrine disruption, sexual behaviour, frog, Key words: Naphthenic acids

## Abstract

Naphthenic acids (NAs), the carboxylic acids found in petroleum, are of emerging concern as they contaminate coastlines after oil spills, leech into freshwater ecosystems of oil sands areas and have wide industrial applications. They are acutely toxic in fish and tadpoles and may be endocrine disruptors at sublethal levels. We characterized androgen-dependent courtship behaviours and their disruption by NAs in male Western clawed frogs, *Silurana tropicalis*. Courtship primarily consists of males producing low trills and achieving amplexus, a mating position where a male clasps a female. Adult males were exposed for 5 days to 20 mg/l NA and injected with human chorionic gonadotropin to induce calling. The duration of calling activity was significantly reduced by NA exposure. Other acoustic parameters such as dominant frequency, click rate and trill length were not affected. Vocalization and amplexus were both inhibited after NA exposure and restored after 2 weeks of recovery in clean water. To determine possible disruption at the level of the testes, the effects of NA exposure on gene expression of key players in steroidogenesis was determined. Exposure to NAs decreased *srd5a* on average by ~ 25%. The enzyme 5α-reductase, encoded by *srd5a,* converts testosterone to its more bioactive form 5α-dihydrotestosterone (DHT), so NAs may be affecting this steroidogenic step. However, the observed upregulation of *lhr, star* and *cyp17a1* suggests that NA-exposed males may be attempting to counteract the reduced potential to produce DHT. Yet, these NA-exposed frogs have dramatically reduced calling duration, so the observed upregulation of *star* and *cyp17a1* is decoupled from the vocalizations. Calling duration and the ability of males to amplex females is reversibly disrupted by NA exposure, implying that environmental reduction and removal of NAs may help improve habitability of contaminated ecosystems.

## Teaser

Naphthenic acids (NAs) in petroleum pollute aquatic environments and can disrupt hormone activity. Male frogs exposed to NAs had disrupted testicular function and suppressed mating behaviours, despite appearing otherwise healthy. These effects were reversed after the frogs returned to clean water, suggesting that contaminated ecosystems can be recovered.

## Introduction

Naphthenic acids (NAs) are a diverse group of compounds that encompass all carboxylic acids found in crude oil, accounting for up to 3% of total mass (Derungs, 1956). Consequently, they have been detected in coastal sediments after major oil spills in the USA (McNutt *et al.*, 2012) and South Korea (Wan *et al.*, 2014). NAs originating from crude oil may also be present in refinery wastewater (Wang *et al.*, 2015a) and are significant toxic pollutants resulting from oil sands development (Bauer *et al.*, 2015, 2019a, 2019b; Frank *et al.*, 2021; Headley and McMartin, 2004; Leung *et al.,* 2003). Extraction from sand requires water and solvents, generating oil sands-process affected water (OSPW) as waste. Large volumes of OSPW with NA levels often exceeding 100 mg/l are currently held in artificial tailings ponds. In 2018, total OSPW volumes were ~1.2 trillion litres (Alberta Energy Regulator, 2019). Despite a zero-discharge policy, NAs have been detected in surface water of the Athabasca region of the Alberta oil sands (Frank *et al.*, 2021; Headley and McMartin, 2004; Leung *et al.*, 2003). Various NAs are also used commercially in a wide range of products such as paint and ink driers, wood and fabric preservatives, fuel additives, emulsifiers and surfactants, and are used in the production of metal salts (Brient *et al.,* 1995). Considering that over 100 countries produce petroleum, the possible consequences of NAs released to the environment through mining and commercial products are of international concern.

The toxicity of NAs has largely been studied in fish models including zebrafish (Scarlett *et al.*, 2013), fathead minnows (*Pimephales promelas*) (Kavanagh *et al.*, 2012) and walleye (*Sander vitreus*) (Marentette *et al.*, 2015). These studies have estimated several LC50s (median lethal concentrations) ranging from 5 to > 50 mg/l depending on the source of NAs. At sublethal levels, some NAs act as endocrine disruptors, especially as strong androgen antagonists (Thomas *et al.,* 2009). Goldfish (*Carassius auratus*) caged in OSPW containing large amounts of NAs showed elevated plasma cortisol, indicating an endocrine stress response. Plasma levels of testosterone were also reduced in both male and female goldfish (Lister *et al.,* 2008). In fathead minnows, a 21-day exposure to an NA extract impaired reproduction (Kavanagh *et al.*, 2012). The NAs decreased fecundity in females and inhibited the development of secondary sex characteristics in males. These changes were likely the result of lowered plasma concentrations of sex steroids, especially androgens, which regulate expression of reproductive behaviours and spermatogenesis (Munakata and Kobayashi, 2010). On the other hand, not all OSPWs containing NAs inhibit fathead minnow reproduction (Morandi *et al.,* 2020). In addition to anti-androgenicity, NAs may be weakly estrogenic (Thomas *et al.*, 2009). This activity may be partly attributed to structural similarities to estradiol and estrone as gas chromatography–mass spectrometry data suggest the presence of steroid-like aromatic NAs (Reinardy *et al.*, 2013; Rowland *et al.,* 2011). Potential oestrogen-like activity has been demonstrated in larval zebrafish, with NAs upregulating gene expression for oestrogen receptor-alpha and vitellogenin (Wang *et al.,* 2015b). In more recent studies, it has been demonstrated that toxicity is associated with not only NA-like O_2_ compounds but also >O_3_+ poly-oxygenated compounds in bitumen-affected water (Bauer *et al.*, 2019b).

To date, amphibian studies have largely focused on NA toxicity and sublethal effects in larvae. Tadpoles of Northern leopard frogs *(Lithobates pipiens)* and Western clawed frogs (*Silurana* (*Xenopus*) *tropicalis*) experience 100% mortality after 24-h exposure to 6 mg/l of a commercial NA preparation (Melvin and Trudeau, 2012a). The 72-h LC50 was estimated at 4.1 mg/l NA for *L. pipiens* and 2.95 mg/l NA for *S. tropicalis*. Exposed tadpoles displayed irregular convulsive swimming and physical abnormalities, such as bent tails (Melvin and Trudeau, 2012a). After chronic exposure to low, environmental doses, *L. pipiens* tadpoles showed reduced glycogen stores and increased triglycerides, indicating disrupted liver and metabolic functions (Melvin *et al.*, 2013). Tadpoles of the marsh frog *(Rana ridibunda)* exposed to water-soluble fractions of crude oil from Kazakhstan experienced reduced growth, delayed development, disrupted biochemical pathways and increased mortality (Sutuyeva *et al.,* 2019). Similar effects are demonstrated in tadpoles of wood frogs *(Lithobates sylvaticus).* They experience reduced growth, an LC50 of ~ 3–4 mg/l, and 100% mortality at 6 mg/l total NAs (Melvin and Trudeau, 2012b). The EC50 estimates based on frequencies of developmental abnormalities in *S. tropicalis* larvae were 2–14 mg/l for various NA extracts. The main effects observed were reduced body size, edema and cranial, heart, gut and ocular abnormalities (Gutierrez-Villagomez *et al.*, 2019b). From these studies, the LC50, EC50 and EC20 values for *S. tropicalis* embryos (Gutierrez-Villagomez *et al.*, 2019b) are less than or within the concentration ranges reported in some OSPW and natural groundwater samples in Alberta. Global transcriptomic responses in whole *S. tropicalis* embryos indicated that numerous gene pathways related to gut function, edema and cartilage development are disrupted by sublethal levels of NAs (Gutierrez-Villagomez *et al.*, 2019a). Furthermore, these data suggest that NAs have multiple modes of action to induce developmental effects in amphibians. Based on this transcriptomic analysis, it can be concluded that some modes of action may be shared between commercial NAs and those extracted from OSPW (Gutierrez-Villagomez *et al.*, 2019a). Similar conclusions were drawn when comparing transcriptomic responses of larval flathead minnows exposed to various NA sources (Loughery *et al.*, 2019).

Given the potential of sublethal concentrations of various commercial and industrial sources of NAs to cause developmental and endocrine disruption, we asked whether NAs may also disrupt reproduction in the African Western clawed frog *S. tropicalis,* a diploid model species commonly used in developmental biology and toxicology (Rosenfeld *et al.,* 2017). To accomplish this, hormone-dependent male vocalizations and courtship behaviours were quantified after exposure to a commercial NA preparation. The important role of androgens in male vocalization and reproduction has been well established in frogs. In the Southern clawed frog (*Xenopus laevis*), amplexus and vocalization can both be induced by human chorionic gonadotropin (hCG), an analogue of pituitary luteinizing hormone (LH) that triggers gonadal steroid production. In castrated adult males, hCG no longer promotes calling. However, exogenous testosterone (T) and its active androgenic metabolite 5α-dihydrotestosterone (DHT) both restore calling in castrated males, and laryngeal muscles are dense in androgen receptors, demonstrating that the behaviour is dependent on testicular androgens in *X. laevis* (Wetzel and Kelley, 1983). Therefore, using targeted measurements of gene expression related to sex steroid synthesis, we also determined whether NA exposure affected testicular function.

## Material and Methods

### Animals

All frogs were bred and raised in captivity at the University of Ottawa Aquatics Facility. Adults were fed Nasco frog brittle three times a week while housed in 27-litre aquaria with flow-through filters in groups of up to 40. The frogs were kept at 26°C in a room with a 12:12 h light/dark cycle. Sexually mature males (age 1–4 years) were identified by the presence of black stripes on the forearms (nuptial pads), a secondary sex characteristic.

### Exposure to NAs

Treatment solutions were generated by diluting commercial NAs (Sigma-Aldrich, Cat# 70340, lot no. BCBK0736V) in filtered, dechlorinated water from the University of Ottawa Aquatics facility. Frogs were individually exposed in static glass tanks containing 3 litres of the treatment solution at 26 ± 1°C. Solutions were fully replaced on the third day. For consistency, all experiments used the same batch of the commercial NA extract that we have chemically and biologically characterized extensively (Gutierrez-Villagomez *et al.,* 2017; Gutierrez-Villagomez *et al.,* 2020). Range finding studies (not shown) were conducted to determine a sub-sublethal, environmentally realistic dose at which the adult frogs would not experience overt toxic effects. This was found to be 20 mg/l. After a 5-day NA exposure, all frogs behaved similarly to controls: actively swimming, able to maintain equilibrium and responsive to visual and physical stimuli. To confirm this, swimming speed was measured by gently prodding the hind leg with a pipette tip to generate a swimming burst. A swimming burst is defined by the frog starting in a static position, fully extending the hind legs to propel forwards, and coming to a stop without being stopped by the sides of the tank. Swimming trials were filmed from bird’s eye view with a 1-cm grid placed under the tank. Blind observers analysed the number of grids crossed using BORIS (https://www.boris.unito.it), a video analysis software. Mean swimming speed was not different between control (11.5 cm/s) and NA-exposed (12.8 cm/s) male frogs (*n* = 11, *t* = 0.8390, *P* = 0.41). The experimental NA exposure level is below levels found in tailings ponds and is within the range detected in some groundwater samples and in natural and constructed wetlands in the Athabasca oil sands region of Alberta, Canada. For example, OSPW NA concentrations may be as high as 128 mg/l (McQueen *et al.*, 2017). The maximum concentration in groundwater has been reported in some cases up to 48 mg/l and in fresh water up to 0.7 mg/l (Frank *et al.*, 2014; Grewer *et al.,* 2010). In containment ponds at industrial sites, total NA levels vary from 24.4 to 51.5 mg/l (Frank *et al.*, 2016). In a recent study, total NA fraction compounds (NAFC) were reported to be between 5 and 14 mg/l in wetlands in the oil sands region (Vander Meulen *et al.*, 2021). Over a 4-week experimental period, industrial OSPW containing ~ 75 mg/l NAFC degraded to ~40–50 mg/l total in constructed wetlands (Simair *et al.*, 2021). Therefore, we conducted exposures to 20-mg/l NA.

### Vocalization assays

Experiments were conducted in a 28°C room on a 12:12 h light/dark cycle. Water temperature was maintained at 26°C. With a lack of seasonal environmental signals under laboratory conditions, vocalization is induced following a standard breeding protocol. Frogs were injected in the lymph sac with a priming dose of 25 IU hCG (Millipore, cat. no. 230734), followed by a boosting dose of 100 IU after 1 day. Injections were prepared by dissolving the hCG in 0.7% saline for a volume of 50 μl per injection. After the boosting injection, the frogs were monitored to screen out non-calling individuals. After 5 days of recovery, individuals were exposed to NAs for 5 days. At the beginning of the sixth day, calling was induced with hCG and recorded. A randomized block design was used, that is, at least 1 frog from each treatment was recorded simultaneously to account for potential effects of time and potential uncontrollable variables (e.g. microclimate fluctuations, unexpected disturbances from surrounding rooms). Tanks were visually and acoustically isolated with pyramid foam. A hydrophone (Aquarian Hydrophones, H2A-XLR) was placed in each tank and used in combination with an external interface (UMC404HD) plugged into a computer.

**Figure 1:**
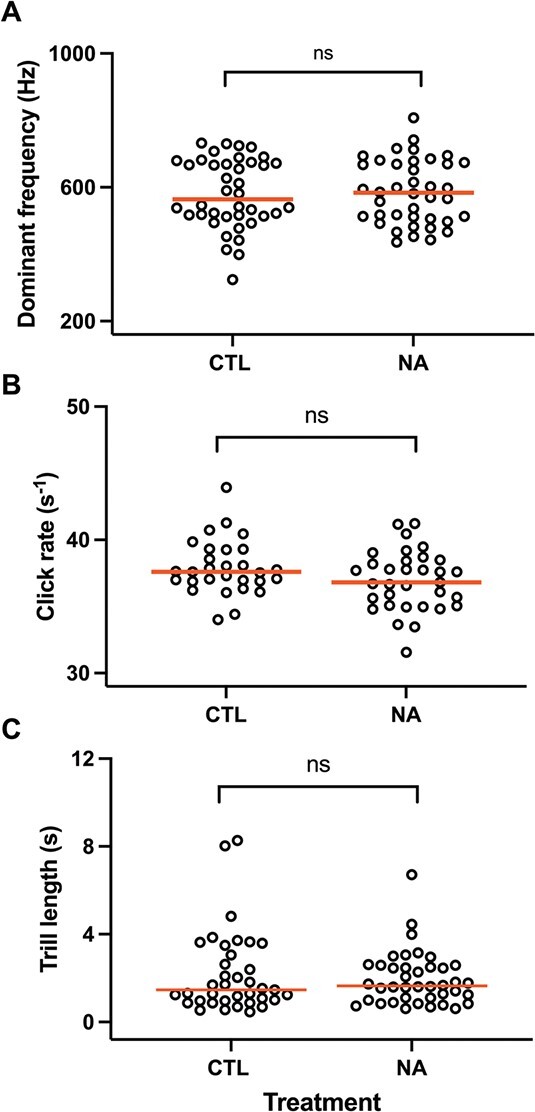
Effect of NA exposure on acoustic properties of vocalizations: (A) dominant frequency, (B) average click rate or (C) average trill length. Individual data presented and pooled across all experiments with red lines at medians. No significant (ns) effects were found (*P* > 0.05).

### Audio analysis

Calls were recorded with multichannel recording software (MixPad, NCH Software) and exported to audio editing software (Audacity, Version 2.4.2) to visualize sound waveforms and spectrograms. Audio files were analysed by several blind observers, with a total of 36 files analysed by two different people. The inter-observer repeatability, that is, the correlation between the values obtained by two observers for the same file was very high (*n* = 36, *r* > 0.99). Absolute calling duration was measured by summing the duration of each trill. Although there was no quantitative definition of a trill or inter-trill intervals, individual trills are visually and acoustically distinguishable with a clear start and end. Spectral plots were generated by Fast Fourier Transform on pooled calls from an individual frog. The frequency of the highest peak was recorded, obtaining one dominant frequency per frog. Click rate was manually counted over time and averaged over five randomly selected trills from each frog. The four types of *S. tropicalis* calls were observed (Fig. 1) and consistent with previous descriptions (Miranda *et al.,* 2015). However, in our animals, slow trills were very rare and not observed in most frogs. Clicks are difficult to distinguish from background noise; therefore, only fast trills were considered for the analyses. This general experimental plan was repeated five times with modifications. In February 2018 (*n* = 7) and May 2018 (*n* = 12), frogs were screened 5 days before the experiment. Since all frogs called, the screening step was not conducted in May 2019 (*n* = 8), nor in the remaining experiments. Given that NAs supress calling in our frogs (see Results) and hCG induces it, we reasoned that perhaps high doses of hCG could be used to enhance testicular function and thus rescue calling in NA-exposed males. In September 2019 (*n* = 8), control and NA-exposed males were given the normal priming dose and a boosting dose of 100 IU hCG after 1 day, whereas other groups of NA-exposed males were given higher boosting doses of 300 or 600 IU hCG in an attempt to hormonally rescue the NA-inhibited calling behaviours. For all experiments described thus far, naïve frogs were used.

In March 2020 (*n* = 10), another vocalization assay was conducted and these frogs were kept for further testing. To examine effects of NA exposure on mate competition, pairs of one control and one NA-exposed male were rinsed and placed in 3-litre clean water to remove surface NAs immediately after the vocalization assay. After 1 h, the pair of males was allowed to freely interact with a sexually receptive female who had been hCG-injected according to the same protocol and was ovulating. The trio was left undisturbed until the female was in amplexus with one of the males. Afterwards, the same male frogs from the competition experiment were maintained under normal, NA-free conditions for 14 days, then retested to determine if the inhibitory effects of NAs are reversible.

### Tissue sample collection and qPCR

In the May 2019 vocalization assay, testicular tissue was harvested and analysed for expression of key genes controlling sex steroid production. An additional set of males was concurrently exposed and injected with saline, generating four treatment groups: control exposure + saline injection, control exposure + hCG injection, NA exposure + saline injection and NA exposure + hCG injection. Immediately after audio recording, frogs were anaesthetized in buffered 1-g/l tricane mesylate (TMS). Testes were dissected with the order of sample collection alternated between treatments. Samples were frozen in dry ice and stored at −80°C for later analysis.

Total RNA was isolated from a single testis (5.9–16.5 mg) for each frog (*n* = 8 per group) according to manufacturer’s instructions for the RNeasy Mini Kit (QIAGEN). Extracted RNA was re-suspended in 30 μl of RNase free water. NanoDrop ND-2000 (Thermo Scientific) was used to determine total RNA concentration as well as the purity of extracted RNA by measuring nucleic acid absorbance ratios *A*_260/280_ and *A*_230/260._ Extracted RNA integrity as assessed using a 1% (w/v) agarose electrophoresis gel containing 1% SYBR Safe DNA stain (Thermo-Scientific) at 90 V for 60 min. All RNA samples were stored at −80°C until used for PCR.

Synthesis of complimentary DNA (cDNA) was performed with the cDNA Maxima Kit (Thermo-Scientific) in a total reaction volume of 20 μl. All RNA concentrations were standardized to 125 ng/μl prior to reverse transcription and each reaction contained 1 μg of total RNA for conversion into cDNA. The cDNA synthesis thermal cycling was performed in the Eppendorf MasterCycler with the following protocol: 10 min at 25°C followed by 30 min at 50°C and a final deactivation cycle for 5 min at 85°C. Samples were stored at −20°C.

Quantitative real-time PCR (RT-qPCR) primers (Table 1) for six biologically relevant genes (*lhr, star*, *cyp17a1*, *cyp19*, *srd5alpha2* and *srd5beta*) and one housekeeping gene (*rpl8*) were designed using NCBI Primer BLAST (https://www.ncbi.nlm.nih.gov/tools/primer-blast/) and synthesized by Integrated DNA Technologies. The cDNA templates were amplified using Maxima SsoAdvanced SYBR Green qPCR Master Mix (Thermo-Scientific) following the manufacturer’s instructions. All PCR products were sequence-verified. All samples were run in triplicate using 200-μl tubes. Each 20-μl reaction consisted of 5 μl of cDNA template, 1× SsoAdvanced SYBR Green Master Mix, 500 nM each of forward and reverse primers and nuclease-free water. All RT-qPCR reactions were completed using the BioRad CFX96 qPCR system with the following thermal cycling parameters: an initial Taq polymerase activation step for 30 s at 95°C, then 40 cycles of a 10-s denaturation step at 95°C followed by a 30-s annealing step at 60°C. A 2-fold, seven-point standard curve was run for each gene in tandem with sample unknowns to verify reaction efficiencies (92.4%–100.5%; *r*^2^ = 0.99). Relative mRNA levels were calculated based on Cq values using the relative standard curve method for RT-qPCR. The data were normalized using the NORMA-Gene algorithm (Heckmann *et al.,* 2011).

### Statistical analysis

Analyses were conducted using Graph Pad Prism (Version 8.0) and *R* (Version 3.6.0). All *P* values are two-tailed with α = 0.05. Normality of residuals was tested using the Shapiro–Wilk test and visualized with QQ plots. Homogeneity of variances was tested using Levene’s test and visualized with residual plots respectively. Calling duration between groups was compared with Kruskal−Wallis ANOVA or Mann–Whitney *U* test on ranks as the data were not parametric. The exception is the data from the May 2018 assay that were parametric and analysed with Student’s *t* test. The latency data from the rescue experiment were analysed with log-rank comparison of survival curves. Frogs who never called were censored and assigned a latency of 5400 s (the length of the experiment). Recovery of vocalization was analysed with two-way repeated-measures ANOVA with subject and replicate as random effects and time (after exposure or after recovery) and treatment as fixed effects. The mate competition data were analysed using a binomial probability test, comparing results to 0.5 probability of success expected by random chance.

All data of individual recordings after exposure were pooled: the vocalization assays, the CTL + 100 IU hCG and NA + 100 IU hCG of the rescue experiment and vocal data from the competition experiment before recovery. The data were normalized by experiment with the lowest value as 0 and the highest value as 1 and analysed with Mann–Whitney *U* test on ranks.

The gene expression data were analysed as two-way ANOVAs with exposure (control or NA) and injection (saline or hCG) as the two factors. The data for *lhr, srd5a, srd5b, cyp17a1* and *cyp19a1* were log(x + 1) transformed to meet assumptions of normality and homoscedasticity.

## Results

### Description of vocalizations in male *S. tropicalis*

The four types of male *S. tropicalis* calls previously reported (Miranda *et al.*, 2015) were all observed and further characterized (Fig. 1). Slow trills were very uncommon and were not produced by most frogs. Clicks resembled background noise, and accurate durations were also difficult to obtain as they usually lasted < 0.1 s. Males generally start vocalizing ~30 min after the second injection of hCG, and some continued calling over 24 h later. The frogs produce several frequencies with an average of 584 Hz (*n* = 80; range, 324–808). The pooled averages were 581.4 and 586.1 Hz for control and NA, respectively (*n* = 40, *t* = 0.21, *P* = 0.83). The trills consist of rapid clicking with an average rate of 37 clicks/s (range, 31–44). The pooled averages were 37.9 and 36.9 for control and NA, respectively (*n* = 25 and 33, respectively; *t* = 1.8, *P* = 0.08). The average trill length for each frog was calculated as total duration divided by total number of calls and ranged from 0.5–8.3 s/call (*n* = 37 and 40, respectively; median = 1.5 and 1.6 s/call for control and NA-exposed, respectively; U = 726, *P* = 0.89). No significant differences were found for these parameters (Fig. 1).

### Disruption and rescue of vocalization

**Figure 2:**
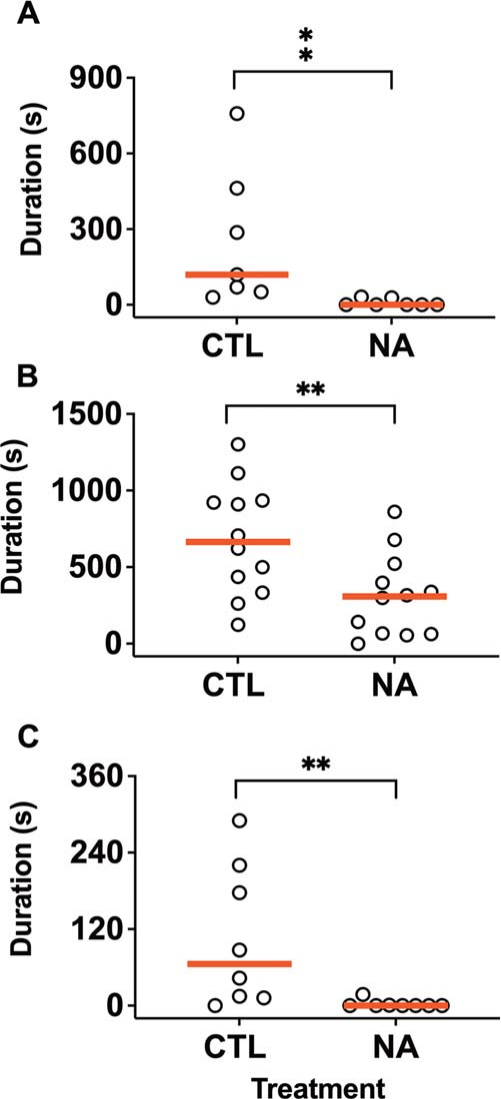
Effect of NA exposure on total calling duration in (A) February 2018, (B) May 2018 and (C) May 2019. Individual data presented with red lines at medians. Statistical significance indicated by asterisks. ^**^P < 0.01

**Figure 3:**
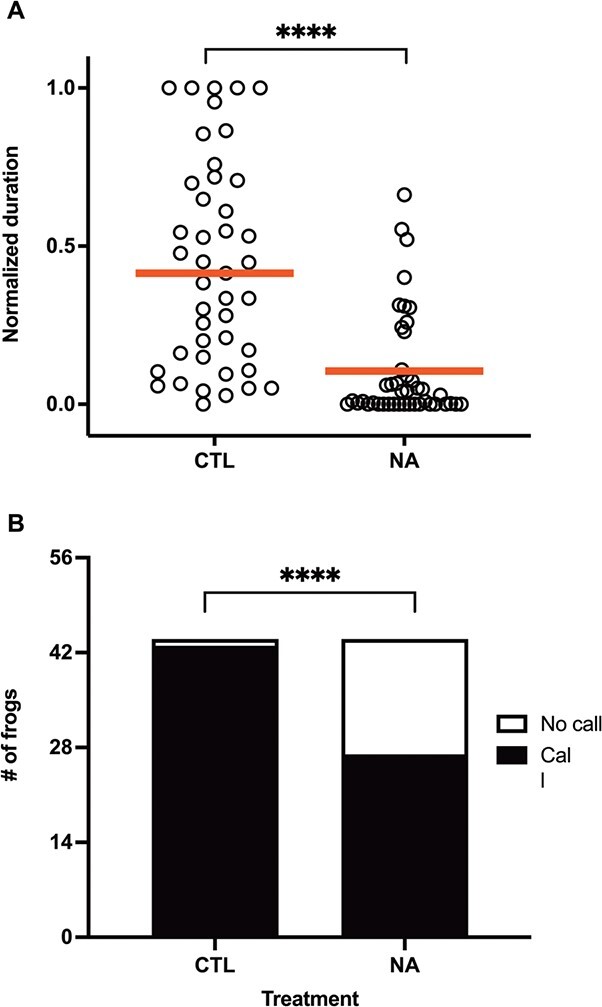
Effect of NA exposure on total calling duration, pooled across all experiments. (A) Individual data normalized proportionately from 0 to 1 and with red lines at medians and (B) count data of calling and non-calling frogs. Statistical significance is indicated by asterisks. ^****^P < 0.0001.

**Figure 4:**
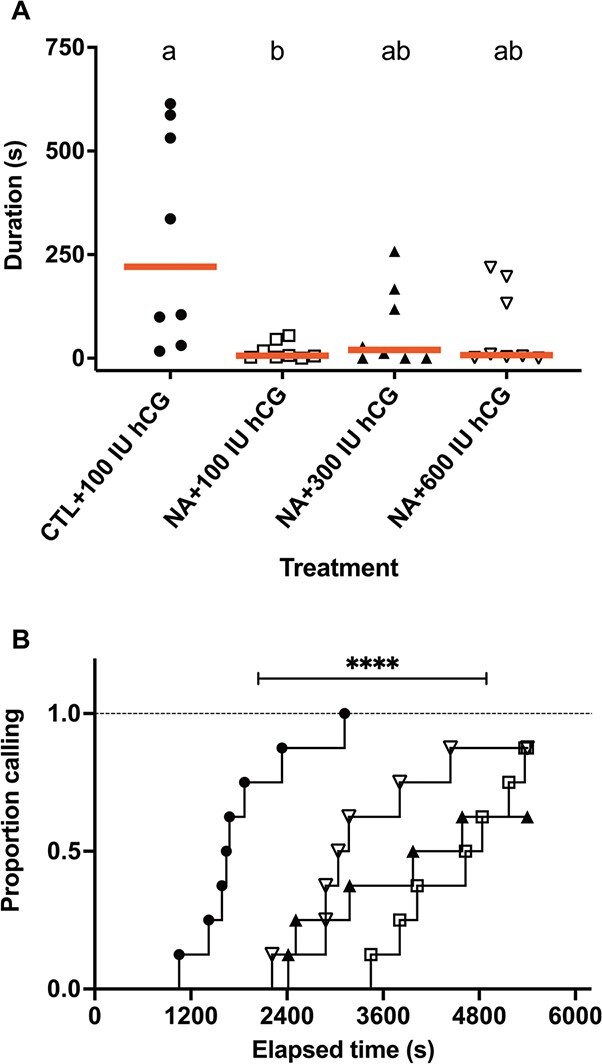
Effect of NA exposure and hCG rescue on calling duration and latency to call. (A) Individual total calling duration data are presented with red lines at medians. Different letters represent statistically significant difference (*P* < 0.05). (B) Individual latency data are presented as survival curves. Statistically significant difference is indicated by asterisks. ^****^P < 0.0001.

In contrast to the acoustic properties of vocalizations, vocal output was clearly lower in the NA-exposed frogs in all three experiments (Fig. 2). The effect sizes varied in scale and magnitude between experiments due to differences in age and cohort of the males used. Median calling durations were 119.7 and 0 s in February 2018 (*n* = 7, U = 1, *P* = 0.001), 664.2 and 307.6 s in May 2018 (*n* = 12, *t* = 2.8, *p* = 0.009) and 65.27 and 0 s in May 2019 (*n* = 8, U = 7, *P* = 0.006). Pooling normalized individual vocalization data from all experiments (Fig. 3A) confirms that NA exposure reduce vocal output (U = 115, *P* < 0.0001). In total, 43/44 (98%) control frogs called, while 27/44 (61%) NA-exposed frogs called (Fisher’s exact *P* < 0.0001) (Fig. 3B). In the hCG rescue experiment (Fig. 4), the control + 100 IU hCG, NA + 100 IU hCG, NA + 300 IU hCG and NA + 600 IU hCG groups had median calling durations of 220.9, 6.1, 19.9, and 7.6 s, respectively, which overall was different between groups (KW = 8.7, *P* = 0.03). Dunn’s multiple comparisons show that calling duration was lower in the NA-exposed frogs than control (*P* = 0.04), consistent with previous assays (Fig. 4A). Injection of both higher doses of hCG appeared to increase calling duration in 3/8 males. However, on average calling durations were intermediate in the NA + 300 IU hCG, and NA + 600 IU hCG groups and were not significantly different from controls (*P* = 0.17 and 0.2, respectively). All three NA-exposed groups were not different from each other (*P* > 0.99). Controls start calling after ~30 min (median latency = 1662.5 s), much earlier than the NA, NA + 300 IU hCG and NA + 600 IU hCG groups (median latency = 4732.66, 4279.47, and 3105.84 s, respectively). Log-rank test shows an overall difference in latency between groups (X^2^ = 34.3, *P* < 0.0001). Although there appears to be a dose-dependent rescue effect, the difference between the NA groups is not significant (X^2^ = 3, *P* = 0.2) (Figure 4B).

### Disruption and recovery of mating and vocal behaviour

**Figure 5:**
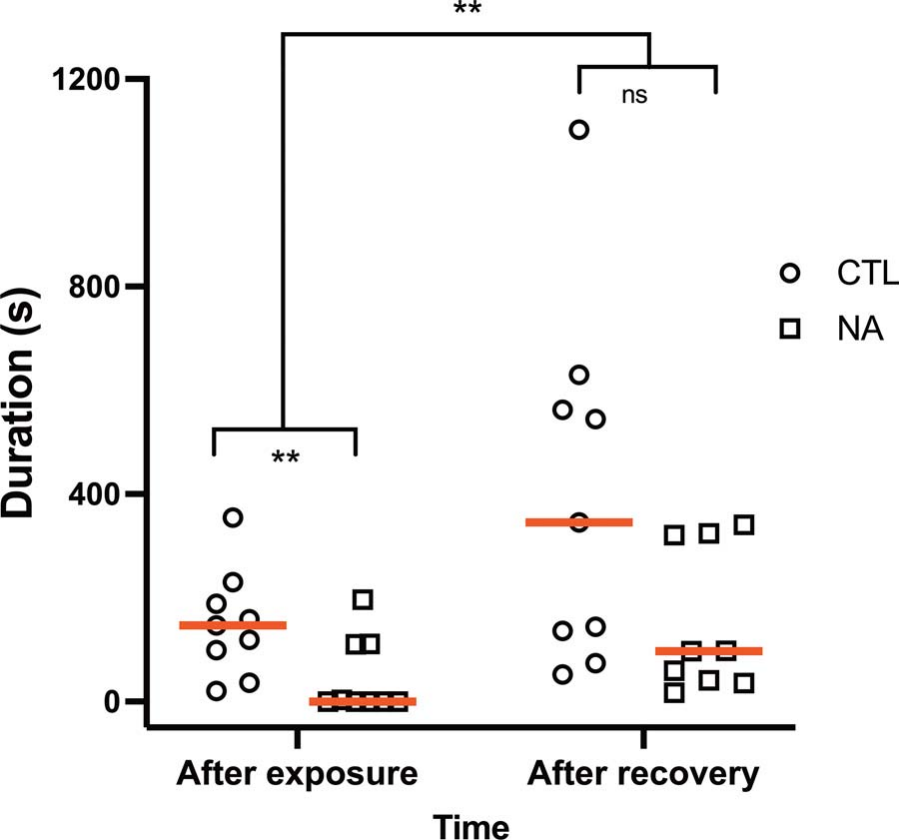
Effect of NA exposure and two weeks of recovery in clean water on individual calling duration. Analyses performed on log(x + 1) transformed data. Raw untransformed data are presented with red lines at medians. Statistical significance is indicated by asterisks. ^**^P < 0.01, ns; not statistically significant.

Vocalization in NA-exposed frogs was assessed by comparing vocal output before and after recovery. The fixed effects used in the model were treatment, time (after exposure and after recovery) and their interaction. There were significant effects of time, treatment and interaction (*F*_1,36_ = 11.63, *P* = 0.0016; *F*_1,36_ = 8.3, *P* = 0.007; and *F*_1,36_ = 4.9, *P* = 0.03, respectively) (Fig. 5). Tukey’s post-hoc shows that controls had higher vocal output than exposed frogs after exposure (*P* = 0.005), which is consistent with all previous vocalization assays. After recovery, the NA-exposed frogs called more than they did before (*P* = 0.005) and were no longer different from controls (*P* = 0.9). The random effect of individual was not significant (*P* = 1), and the results did not change when it was excluded from the model.

**Figure 6:**
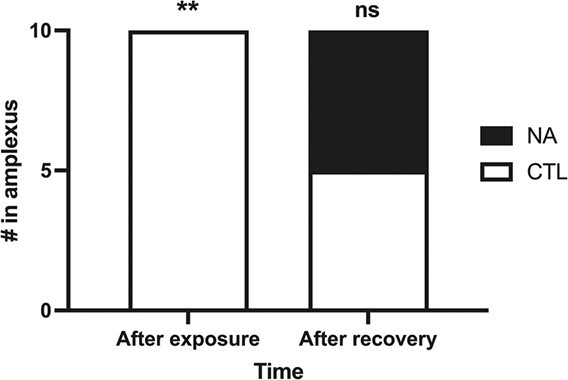
Mating outcome of pairs of a control and exposed male with one female after exposure and after recovery. Count data are presented. Separate analyses were performed for after exposure and after recovery. Asterisks indicate statistical significance. ^**^P < 0.01, ns; not statistically significant.

After audio recording, mate competition was tested as the two males were allowed to freely interact with a sexually receptive female. In the first set of trials following the NA exposure, the exposed males failed to amplex the females in 10/10 trials (*z* = 2.85, *P* = 0.001). In the second set of trials after 2 weeks of recovery in clean water, both control and NA-exposed groups of males both amplexed females in 5/10 trials (*z* = 0, *P* = 0.25) (Fig. 6).

### Effect of NA exposure on hCG-induced testicular gene expression

**Figure 7:**
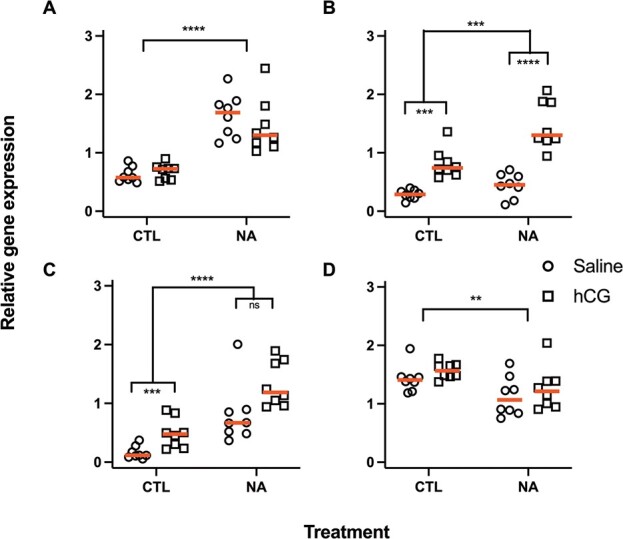
Relative gene expression of (A) *lhr*, (B) *star*, (C) *cyp17a1* and (D) *srd5a*. Raw untransformed data are presented with red lines at medians. Statistical significance is indicated by asterisks. ^**^P < 0.01, ^***^P < 0.001, ^****^P < 0.0001, ns; not statistically significant

Levels of *lhr* mRNA (Fig. 7A) were not affected by hCG injection (*F*_1,28_ = 0.1, *P* = 0.8). However, exposure to NA upregulated *lhr* (*F*_1,28_ = 107.4, *P* < 0.0001), with no significant exposure × injection interaction evident (*F*_1,28_ = 1.8, *P* = 0.2). Expression of *star* (Fig. 7B) was significantly affected by hCG injection, NA exposure and their interaction (*F*_1,28_ = 71.2, *P* < 0.0001; *F*_1,28_ = 19.3, *P* = 0.0001; *F*_1,28_ = 7.3, *P* = 0.01). Results from Sidak’s multiple comparisons test showed that both hCG injection and NA exposure upregulated *star* and that expression was higher in NA-exposed frogs than controls within the hCG injection groups (*P* < 0.0001). Expression of *cyp17a1* (Fig. 7C) was increased by both NA exposure and hCG injection, but with no interaction effect (*F*_1,28_ = 58.8, *P* < 0.0001; *F*_1,28_ = 24.4, *P* < 0.0001; *F*_1,28_ = 2.6, *P* = 0.1). Expression of *srd5a* (Fig. 7D) was lower in NA exposed frogs, but not affected by injection or interaction (*F*_1,28_ = 11.3, *P* = 0.002; *F*_1,28_ = 2, *P* = 0.2; *F*_1,28_ = 0.01, *P* = 0.9). For *cyp19a1* and *srd5b,* there were no significant effects (data not shown).

## Discussion

We report for the first time the suppressive effects of NAs on breeding behaviours in a frog. Adult male *S. tropicalis* were resistant to overt toxicity of NAs, considering the high sensitivity of their larvae. Larval *S. tropicalis* exhibit a LC50 of 11.7 mg/L NA after 96 h of exposure to the same commercial NA product (Gutierrez-Villagomez *et al.*, 2019b). In the experiments presented, males were exposed to 20 mg/l for 5 days and still appeared healthy. However, numerous males exposed to the NA mixture did not vocalize in response to hCG, or if they did, call duration was significantly reduced. Although the exposed frogs have lower absolute calling activity, the call characteristics (pitch, click rate and trill length) do not appear to be affected by NA exposure. As these acoustic parameters depend on larynx morphology and muscle mechanics, NAs may be acting on the hormonal triggers of vocalization rather than physically damaging larynx tissues. There is indication that the effects are reversible to some extent, since call durations after a 2-week recovery period after the 5-day NA exposure were similar to control values.

Considerable data indicate that male vocalizations in anurans are dependent on testicular androgens. Calling is inhibited by castration and restored with exogenous androgens in species such as *X. laevis* (Wetzel and Kelley, 1983) and the green tree frog *(Hyla cinerea)* (Burmeister and Wilczynski, 2001). Calling and clasping can be induced by injecting homogenized whole pituitaries in male leopard frogs (*Lithobates pipiens),* leading to a sustained increase in plasma T and 5α-DHT, along with increased calling and clasping behaviour (Wada *et al.,* 1976; Wada and Gorbman, 1977). These behaviours are abolished by castration and restored with T and testicular implants (Palka and Gorbman, 1973; Wada and Gorbman, 1977). Androgens also modulate vocalization through central pathways. Androgen-concentrating cells have been found in the brain along neural pathways involved in vocalization, although these findings are limited to *X. laevis* (Kelley, 1980, 1981; Kelley *et al.,* 1975)*.* Our results on the suppressive actions of NAs in *S. tropicalis* are consistent with the effects of several anti-androgenic EDCs in *X. laevis*. Vinclozolin, a commonly used dicarboximide fungicide with anti-androgenic effects, disrupted male vocalization. Males exposed for 96-h experienced diminished calling activity due to a decrease in utterance of advertisement calls and chirping, indicating sexual non-receptivity (Hoffmann and Kloas, 2010). Exposure to flutamide, a selective antagonist of the androgen receptor, also reduced advertisement calls (Behrends *et al.,* 2010). Opposite effects were produced with the potent androgen agonist methyl-dihydrotestosterone (Hoffmann and Kloas, 2012).

We also investigated the effects on NA exposure on gene expression to determine possible disruption at the level of the testes. The LH analogue hCG activates gonadal LH receptors to increase steroid production and subsequently induce the full suite of male and female behaviours leading to successful spawning in frogs such as *X. laevis* and *S. tropicalis*. Expectedly, hCG effectively induced calling in control males and we observed the expected increase in the expression of testicular *star* and *cyp17a1*. On the other hand, hCG did not affect *lhr* or *srd5a*. Surprisingly, NA-exposed frogs showed higher levels of *lhr*. Moreover, hCG-induced *star* levels were enhanced by NA exposure, perhaps due to upregulated LH receptors. Both hCG injection and NA exposure upregulated *cyp17a1*, a major enzyme in androgen production. Exposure to NAs decreased *srd5a* on average by ~ 25%. The enzyme 5α-reductase, encoded by *srd5a,* converts testosterone to its more bioactive form 5α-DHT, so NAs may be affecting this key steroidogenic step. On the other hand, the upregulation of *lhr, star* and *cyp17a1* in NA-treated males may be compensatory, and the males may be attempting to counteract the reduced potential to produce 5α-DHT. Yet, NA-exposed males have dramatically reduced calling duration, so the observed upregulation of *star* and *cyp17a1* is somehow decoupled from the vocalizations. Thus, the consequences of low 5α-reductase activity should not be overlooked, given its paramount importance in male reproduction (Robitaille and Langlois, 2020).

We reasoned that administration of higher doses of hCG may be able to override the inhibitory actions of NAs on calling behaviour. In this experiment, total calling duration was again dramatically reduced in the NA-exposed frogs, and 3- and 6-fold higher doses of hCG did not rescue the supressed vocalization activity. Control frogs began vocalizing after ~30 min following injection, consistent with previous experiments. Increasing hCG appeared to partly restore latency to control levels, although the difference is not significant. This suggests that the ability of NA-exposed males to respond to LH is impaired. In addition to reduced *srd5a,* there are other possible mechanisms by which NAs could disrupt vocalization. While we did not test these, a couple are worthy of consideration in future experiments. First, in *X. laevis*, LH receptors, the target for hCG action, are expressed in regions of the forebrain controlling vocalization. Indeed, direct injection of hCG into the brain of castrated males treated with androgens provokes calling activity (Yang *et al.,* 2007). Thus, it will be important to determine the effects of NAs on neural LH receptors. Second, the NAs could also be reducing androgen receptor abundance in laryngeal muscles or in forebrain neurons that control calling behaviour (Kelley, 1980).

Mate competition experiments were conducted after vocal recordings by allowing a control and NA-exposed male to freely interact with a sexually receptive female. By the time the female was added, most had already started laying eggs (~4-h post-hCG injection) and were therefore highly receptive. Control males would immediately respond to her presence. When the female swam within one body length, the male would orient towards, chase after, and attempt to clasp the female. Within a few minutes of adding her to the tank, the female would be in amplexus with one of the males. The NA-exposed males did not display these behaviours towards the female or make attempts to clasp. Even when the female swam close by or made contact, they would not orient towards or approach her. The control males successfully amplexed the female in all trials but did not necessarily do so by outcompeting the other male, as the exposed males were not observed attempting to mate. We determined that NA-exposed males had normal locomotion and swimming speeds, so this cannot explain and lack of ability to mate with a female. Regardless, suppressed calling duration and a lack of interest in normal, ovulating females indicates disrupted sexual activity in male *S. tropcalis* exposed to NAs for only 5 days, even when injected with high doses of hCG.

Importantly, these suppressive actions of NAs on calling appear to be reversible as after 2 weeks of recovery in clean water: control and previously NA-exposed frogs had similar call durations. Similarly, burbot (*Lota lota*) exposed to the anti-anxiety drug oxazepam showed decreased sheltering behaviour, and the effect was reversed after 5–7 days in clean water (Sundin *et al.,* 2019). In our experiment, after recovery, mate competition was clearly observed as both control and NA-exposed males attempted to clasp the female *S. tropicalis.* From our general observations during the post-recovery test, males physically competed when both attempted to clasp and dislodge the other by kicking. Some amplexus attempts were not successful as the female also dislodged males. As these observations are qualitative, future work may examine and quantify mate competition behaviours in detail in *S. tropicalis*. A similar mating trial was previously conducted with *X. laevis*. Four females, four control males and four males exposed to atrazine, an anti-androgenic herbicide, were left to freely interact overnight (Hayes *et al.*, 2010). Amplexus was achieved almost exclusively by control *X. laevis* males, but the frogs were not continually observed. In the atrazine exposure experiment, it was unclear if the *X. laevis* controls more successfully attracted the females and outcompeted the exposed males, or if the exposed males made fewer attempts to mate.

Advertisement calls are particularly important for mate competition in low-visibility conditions such as turbid ponds at night. However, in the laboratory setting, the males in our experiments were able to quickly locate and approach the female immediately after she was introduced, making attractive calling less important to mating success. Reduced vocal output as we have observed following NA exposure may have more significant effects in natural conditions where individuals would be more dispersed. Females rely heavily on acoustic signals and phonotaxis to locate the best males, whereas in the lab experiment, males physically fought to achieve amplexus. To elucidate the males’ ability to vocally attract females and the ecological consequences of reduced vocal output, males could be physically but not acoustically isolated in an experimental setting so that the females must approach her preferred mate based on song quality and duration.

A limitation of the study is that the NAs used were from a commercial extract, when they can be very complex and heterogenous in natural environments. Despite advances in analytical methods, elucidation of individual components has been largely unsuccessful and hundreds or thousands of chemicals remain uncharacterized (Clemente and Fedorak, 2005). A large body of evidence shows that NAs differ greatly in composition depending on the source (Clemente and Fedorak, 2005). This leads to variation in structure-dependent uptake, toxicity and neuroendocrine disrupting modes of action. Furthermore, the term OSPW NAs refers to acid-extractable organics (AEOs). Because these mixtures are obtained by extracting O_2_ groups, they can contain a large proportion of compounds that are not true NAs but still contribute to toxicity (Grewer *et al.*, 2010; Morandi *et al.*, 2015). As such, commercial NAs generally have stronger effects than AEOs. Toxicity assays in aquatic invertebrates comparing commercially available NA mixtures to NA fractions from tailings ponds found LC50s several times lower in the commercial extracts compared to those from tailing ponds (Bartlett *et al.*, 2017). Larvae of *S. tropicalis* exposed to commercial NAs showed lower LC50s and EC50s compared to larvae exposed to OSPW AEOs but experienced the same types of morphological abnormalities and disruption of gene networks involved in metabolic and developmental processes (Gutierrez-Villagomez *et al.*, 2019a). Larval fathead minnows (*Pimephales promelas*) exposed to fresh and aged NA fractions from OSPW as well as commercial NAs all showed immunome disruptions (Loughery *et al.*, 2019).

As it is non-invasive and cost-effective, passive acoustic monitoring has been proposed as a method of assessing biodiversity and ecosystem health (Desjonquères *et al.,* 2020). Anuran calling behaviour can be another endpoint to consider for evaluating the long-term ecotoxicological impacts of contaminated environments, such as throughout the reclamation of oil sands tailings ponds Incorporating reproduction-relevant endpoints such as those inhibited by NAs into environmental effects monitoring programs and adaptive management strategies would help to reduce impacts of oil sands operations. For example, setting threshold concentration values in reclaimed wetlands and/or holding and weathering of NA mixtures to a point where biological activity has negligible effects on amphibian mating behaviours and tadpole development warrants consideration.

In conclusion, we characterized the courtship behaviours of a popular anuran model species and found that calling duration and the ability to amplex females is reversibly disrupted by NA exposure. Mitigation and reclamation efforts in areas contaminated with NAs, therefore, may render them habitable if NA release to aquatic systems and residual toxicity is reduced and eventually eliminated.

## Funding

This work was supported by Ontario Government Scholarships and Change to Natural Sciences and Engineering research Council of Canada (NSERC) CGS-M (WSZ), NSERC USRA (EF), NSERC Strategic Grants Programme (STPGP 463041-14 to VLT). Also acknowledged with appreciation are University of Ottawa Research Chair programme and Environment and Climate Change Canada through the Canada-Alberta Joint Oil Sands Monitoring Programme for VLT.

## Data Availability

All data generated or analysed during this study are included in this published article.
